# Study of Hybrid Nanoparticles Modified Epoxy Resin Used in Filament Winding Composite

**DOI:** 10.3390/ma12233853

**Published:** 2019-11-22

**Authors:** Chengrui Di, Junwei Yu, Baoming Wang, Alan Kin Tak Lau, Bo Zhu, Kun Qiao

**Affiliations:** 1 School of Mechanical, Electrical & Information Engineering, Shandong University, Weihai 264209, China; dicr0918@sdu.edu.cn; 2 Faculty of Science, Engineering & Technology, Swinburne University of Technology, Hawthorn, Victoria 3122, Australia; aklau@swin.edu.au; 3 Shandong fengjin new energy technology co. LTD, Yantai 264100, China; 4 School of Materials Science and Engineering, Shandong University, Jinan 250061, China; junweiyu@mail.sdu.edu.cn (J.Y.); 201820371@mail.sdu.edu.cn (B.W.); zhubo@sdu.edu.cn (B.Z.)

**Keywords:** epoxy resin, core-shell rubber (CSR), nano-SiO_2_, wet filament winding

## Abstract

Hybrid nanoparticles modified bisphenol A type epoxy/acid anhydride resin system applicable for filament winding forming process was studied using elastic core-shell rubber (CSR) nanoparticles with a large particle size (nearly 100 nm) and rigid nano-SiO_2_ particles with a small particle size (about 16 nm). The formulation, process properties, mechanical properties, thermal properties and microstructure of modified resin and its wound composite were studied. The results suggested that at the content of 10 phr CSR and 2 phr nano-SiO_2_, the resin system achieved optimum comprehensive performance. The viscosity of modified resin system was nearly 1000 mPa·s at 25 °C and service life was over 6 h. The resin tensile strength and modulus were 89 MPa and 3.5 GPa, while flexural strength and modulus reached 128 MPa and 3.2 GPa, respectively. The impact strength was 26.6 kJ·m^−2^, and the glass transition temperature (Tg) reached 145.9 °C. Modified epoxy resin enhanced the mechanical properties of carbon fiber reinforced wound composite. The tensile strength, tensile modulus and interlaminar shear strength were enhanced by 14.0%, 4.56% and 18.9%, respectively, compared with a composite based on unmodified resin. The above test results and scanning electron microscopy (SEM) analysis suggest that the hybrid nanoparticles modified resin system was suitable for carbon fiber wet filament winding products.

## 1. Introduction

Carbon fiber reinforced polymer composite (CFRP) is characterized by its light weight, high strength, having prominent corrosion resistance, designability and easy forming [1]. As a result, CFRP has been extensively applied in industrial areas as novel structural materials. One of the most important forming processes is the filament winding process, which is commonly employed in the mass production of advanced fiber reinforced composites, such as pressure vessels, pipes and shafts [2]. Most of the above products are produced by wet filament winding process, which has special requirements for resin system matrix (e.g., lower viscosity, longer application period, better elongation, higher modulus as well as thermal stability) [3].

Due to the characteristics of the wet filament winding process, an epoxy/acid anhydride resin system has been adopted in most cases. Nevertheless, this kind system exhibits high brittleness and poor impact resistance, directly affecting the product quality. Thus, the epoxy/acid anhydride resin system requires modification to meet the process and toughness requirements. At present, there are many ways to modify epoxy resin, including the addition of a second phase modifier, for instance, rubber particles [4,5], thermoplastic resin [6], and inorganic nanoparticles [7,8,9].

However, these toughening methods have their own weaknesses. To be specific, rubber and some low-molecular thermoplastic resins can significantly reduce the heat resistance and rigidity of modified resins, while macromolecular thermoplastic resins and inorganic particles can noticeably increase the viscosity of modified resins and affect their process properties. Accordingly, the special requirements of winding resin cannot be easily met by adding only one kind toughening agent.

Nano-materials have considerable excellence of specialty (e.g., the quantum size effect and small size effect) [10]. In particular, core-shell rubber nanoparticles (hereafter referred to as CSR nanoparticles) are excellent toughening materials [11,12,13,14]. Its core is rubber, endowing the resin matrix with better tensile properties; accordingly, it can enhance the ability of resin to resist external forces and increase the tearing strength. Nevertheless, the stiffness and temperature resistance of resin matrix may degrade with the adding of elastic rubber-plastic particles. As a resin matrix for carbon fiber winding products, high modulus and thermal deformation temperature are also required to ensure the structural stiffness of winding products. Nano-inorganic rigid particles can absorb some deformation energy in the matrix resin, prevent destructive cracking and toughen the resin, as well as enhance the rigidity, dimensional stability and heat resistance of the resin matrix. Accordingly, we considered adding inorganic nanoparticles together with core-cell nanoparticles, to achieve excellent comprehensive performance of the modified resin.

In this paper, to prepare a higher performance winding resin system and satisfy the requirements of wet filament winding products, the synergetic modification effect of CSR nanoparticles and nano-SiO_2_ rigid particles on epoxy resin was studied.

## 2. Materials and Methods

### 2.1. Materials

The epoxy resin of bifunctional epoxy resin diglycidyl ether of bisphenol A (E-54; Nantong Xingchen synthetic material Co., Ltd., Wuxi, China) was used in this study, with an epoxy value of 0.52–0.56 mol/100g. The CSR toughening agent (Kaneka, MX-154) was provided by Dalian liansheng trading Co., Ltd. (Dalian, China). The curing agent was methyl hexahydrophthalic anhydride (MeHHPA) from Tianjin Nanya chemical Co., Ltd. (Tianjin, China), and the cure accelerator was 2,4,6-tri (dimethylaminomethyl) phenol (DMP-30) from Changzhou Shanfeng Chemical Co., Ltd. (Changzhou, China). Nano-SiO_2_ (Evonik Degussa, R972) was purchased from Shanghai Shengpu information technology development Co., Ltd. (Shanghai, China). Reactive diluent (Double epoxy functional group, home-made) and silane coupling agent (KH-550) were purchased from Dongguan Kangjin new material technology Co., Ltd. (Dongguan, China). Carbon fiber (Toray, T700SC-12K-50C) was purchased from Wuxi lizhou import and export trade Co. Ltd. (Wuxi, China).

**Figure 1:**
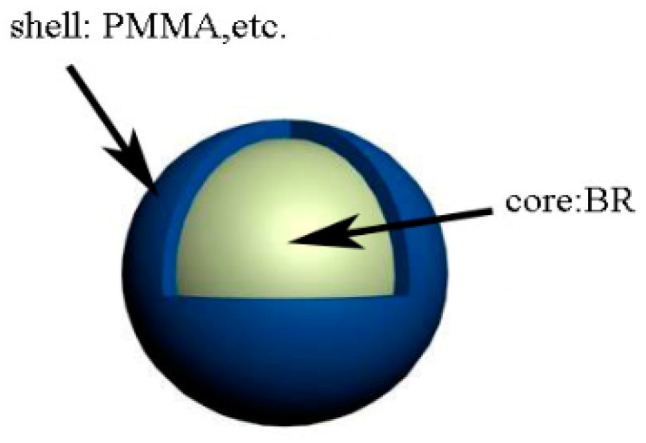
Structure of CSR particle.

The major parameters of CSR nanoparticles and nano-SiO_2_ particles are listed in Table 1 and Table 2 below. The structure of CSR particle is illustrated in Figure 1.

### 2.2. Sample Preparation

#### 2.2.1. Casting of Epoxy Resin

The epoxy resin, curing agent, toughening agent (MX-154), nano-SiO_2_(R972), silane coupling agent and cure accelerator were weighed and then mixed in order, according to the designed proportion of each component. First, at 40–50 °C, the mixture underwent electric mixer mixing at a speed of 800–1000 rpm for 10–15 min; then, the mixture was placed in the ultrasonic chamber and dispersed for 20–30 min. Subsequently, the product was placed in the vacuum oven until there were no bubbles. Afterwards, the blend was transferred into an open stainless-steel mold and then cured in an oven at 90 °C for 2 h, 130 °C for 2 h, and at 150 °C for 2 h in sequence. After being fully cured, the casting body was naturally cooled to ambient temperature.

#### 2.2.2. Composite Samples Preparation

**Figure 2:**
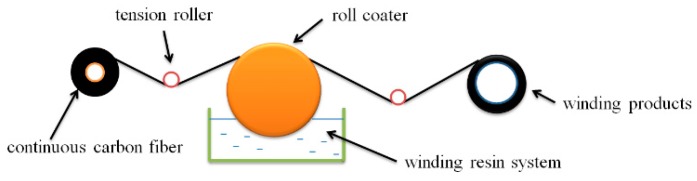
Filament winding process of NOL rings.

The continuous carbon fiber completely soaked in the winding resin was wound to the stainless-steel mold under a tension of 35–40 N using winding machine (type 4FW500 × 4000, Harbin composite material equipment development Co., Ltd., Harbin, China). The winding speed was 30 r/min and the resin temperature in the glue storage tank was 25–30 °C, as shown in Figure 2. After winding, the product was put into the rotary curing furnace, and the curing steps were identical to that of the casting bodies. Lastly, NOL rings (The ring specimen was first used by the Naval Ordnance Laboratory, so it is often called NOL ring) and interlaminar shear specimens were prepared according to GB/T 1458-2008 “test methods for mechanical properties of fiber wound reinforced plastic annular specimens”, and the fiber volume fraction was 57.5%.

### 2.3. Characterization

The tensile strength and modulus, elongation at break, flexural strength and modulus of the cured resin were tested using material testing machine (CMT4204, Shenzhen SANS Testing Machine Co., Ltd., Shenzhen, China) according to ISO 527 and ISO 178, respectively.

The impact strength of the cured resin was tested on a simply supported beam impact testing machine (XJJD-5, Chengde Kaosi Scientific Testing Co., Ltd., Chengde, China) according to ISO 179-1. Besides, the sample size was 80 mm × 10 mm × 4 mm. The impact energy was 2 J, and the impact velocity was 2.9 m/s. Using a thermal deformation vicat softening point temperature tester (XRW-300M, Chengde, China), heat deflection temperature of the resin was tested according to ISO 75-2. The size of sample was 80 mm × 10 mm × 4 mm, and the heating rate was (120 ± 10) °C/h.

The interlaminar shear strength of the NOL ring was ascertained on a short-beam-shear fixture and a short-beam bending test fixture (5966, universal testing machine, Shenzhen, China) following GB/T 1458-2008. The tensile strength of the NOL ring sample was ascertained with a split-disk fixture (WE-10B, universal testing machine, Shenzhen, China) following GB/T 1458-2008.

All the mentioned tests were performed at 23–25 °C, and the reported values were averaged from 6 independent specimens for respective samples.

The glass transition temperature of the samples was ascertained by a dynamic mechanical analysis (DMA) (Q800, New Castle, DE, USA), open air conditions. The frequency was 1 Hz and the heating rate was 5 °C/min.

The viscosity of the epoxy system was ascertained on a rotational viscosimeter according to GB/T7193-2008. The morphology of the fracture surfaces of the cured epoxy resin samples and winding composite samples were observed under scanning electron microscopy (AMARY-1000B, Milpitas, State of CA, USA).

## 3. Results and Discussion

### 3.1. Properties of Modified Epoxy Resin System

#### 3.1.1. Effects of CSR Nanoparticles on the Epoxy Resin System

**Figure 3:**
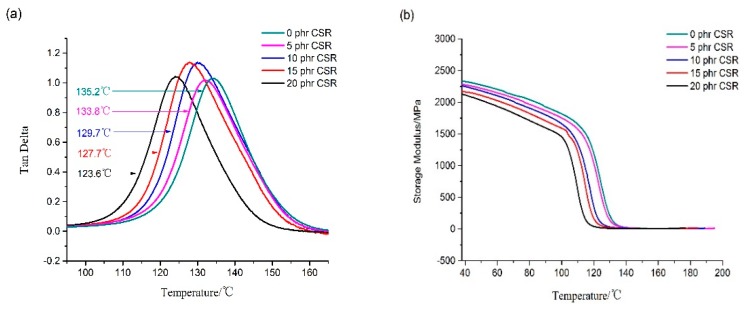
Dynamic mechanical analysis (DMA) test results of CSR nanoparticles modified epoxy resin: (**a**) Tan Delta curves of DMA test; (**b**) Storage modulus curves of DMA test.

The basic composition of epoxy matrix resin covered 100 phr (by weight) epoxy resin, 10 phr active diluent, 85 phr curing agent and 1.5 phr cure accelerator. Besides, the CRS toughening agent was adopted to toughen the epoxy matrix resin. To assess the toughening effect of the amount of toughening agent on epoxy matrix resin, 5 phr, 10 phr, 15 phr and 20 phr of CSR particles were added, respectively. The mechanical properties and thermal properties are shown in Table 3 and Figure 3.

Table 3 suggests that, when CSR particles were added in a certain range, with the increase in CSR particles dosage, the impact strength, tensile strength and elongation at break of epoxy matrix resin grew noticeably. When 20 phr CSR particles were added, the impact strength reached 30.3 KJ·m^−2^, nearly 83.6% higher than that before toughening. When 10 phr CSR particles were added, the tensile strength reached the maximum value of 82 MPa, 30.8% higher than the previous, and elongation at break was increased by 20.9%.

For CSR particles, the predominant toughening mechanisms can be summarized as localized shear-banding of the epoxy polymer initiated by the particles and internal cavitation of the CSR particles. The CSR particles cavitation results in a change in the stress state of the resin, which causes shear yield, prevents further crack growth, consumes a lot of energy, and improves the toughness of the resin [15].

Moreover, under the appropriate amount of CSR, the CSR particles would keep better particle spacing. When the cracks occurred and expanded, the load bearing stress of the CSR particles would hinder or even prevent the growth of the cracks, and the cracks would deflect in the direction near the CSR particles (crack defection, crack branching mechanism) [16]; some cracks would stop (riveted crack mechanism) [17,18].

When the additive amount increased, the CSR particles spacing were too small and even particle agglomeration occurred. The formation of microcracks could be induced due to stress collection [19,20]. As a result, the performance of the resin system would be weakened. Accordingly, as CSR particles continued to increase, the tensile strength and elongation at break decreased slightly (Table 3).

According to the heat deflection temperature in Table 3 and the DMA curves in Figure 3, adding CSR particles would reduce the heat resistance of the resin; the more CSR particles added, the greater the reduction in heat resistance of the resin would be. CSR particles can enhance resin performance, but adversely affect the heat resistance.

In consideration of the mechanical properties, temperature resistance, viscosity and cost of winding resin, it was preferred to add 10 phr CSR particles.

#### 3.1.2. Effects of Nano-SiO_2_ Particles on the Epoxy Resin System

To enhance the heat resistance and modulus of CSR nanoparticles modified epoxy resin, this study continued to add 1 phr, 2 phr, 3 phr, 5 phr of the nano-SiO_2_ particles based on 10 phr CSR modified epoxy resin. Moreover, to improve the compatibility, infiltration and dispersion between the nano-SiO_2_ and resin, 1% coupling agent of the mass of nano-SiO_2_ was added to treat the surface of nano-SiO_2_. Test results of nano-SiO_2_ particles modified epoxy resin were shown in Table 4.

Table 4 suggested that, with the rise in nano-SiO_2_ content, the flexural strength, tensile strength, modulus and impact strength of epoxy matrix resin increased first and then decreased, and the fracture elongation decreased.

When nano-SiO_2_ was added at 2 phr, the tensile strength, flexural strength and impact strength of the resin matrix nearly reached the maximum, and were increased by 10.3%, 6.7% and 5.6%, respectively.

Since the toughening effect of CSR particles is significantly large, the toughening effect of adding nano-SiO_2_ is not noticeable, but helpful to improve the strength and modulus of the resin. Besides the cavitation theory of CSR particles already discussed, the toughening mechanisms of the smaller nano-SiO_2_ particles are mainly crack deflection and branching, riveted crack, which has a synergistic toughening effect with CSR particles [20,21].

In the meantime, the physical or chemical combination of nano-SiO_2_ particles and epoxy resin may enhance the interface bonding, promoting to the stress transfer between particles and the matrix, enhancing the ability to bear the load and increasing the strength of the resin matrix [22]. Due to the large modulus and good heat resistance of nano-SiO_2_ particles, when the particles are added, the crosslinking degree of the resin matrix can increase, and the modulus and heat resistance of the resin matrix can be enhanced [23].

**Figure 4:**
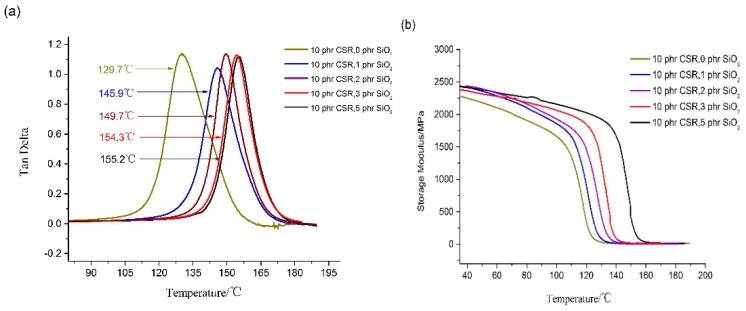
DMA test results of CSR/nano-SiO_2_ modified epoxy resin: (**a**) Tan Delta curves of DMA test; (**b**) Storage Modulus curves of DMA test.

DMA curves of nano-SiO_2_ particles modified epoxy resin are shown in Figure 4, which shows that adding nano-SiO_2_ particles significantly improves the heat resistance of resin. The glass transition temperature of the modified resin increased by 16.2 °C after adding 1 phr nano-SiO_2_ particles. Nevertheless, the glass transition temperature increased little with nano-SiO_2_ particles addition.

However, the results showed that continuing to add more nano-SiO_2_ could cause the strength of epoxy matrix resin to decline, which is consistent with the addition of CSR particles. Likewise, on the base of both consideration of the mechanical properties, viscosity and technology of winding resin, it was preferred to add 2 phr nano-SiO_2_ particles.

#### 3.1.3. The Process Properties of Nano-CSR/Nano-SiO_2_/Epoxy Resin System

The viscosity property of resin system is one of the vital parameters to indicate whether it is suitable to filament winding process.

**Figure 5:**
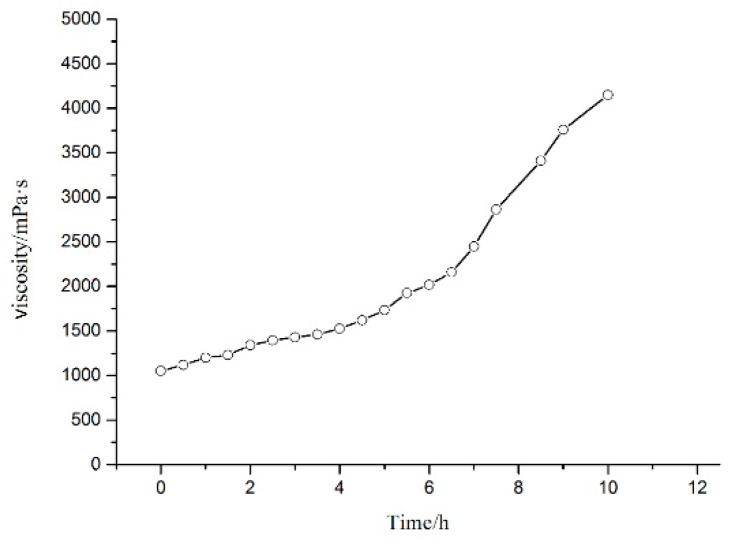
Viscosity–time variation curve of nano-CSR(10 phr)/nano-SiO_2_(2 phr)/epoxy resin system.

The viscosity–time variation curve of nano-CSR (10 phr)/nano-SiO_2_ (2 phr)/epoxy resin system (Figure 5) showed that at 25 °C the viscosity of the resin system was nearly 1000 mPa·s and remained below 1500 mPa·s for nearly 3 h; after 6 h, the viscosity of the resin system reached 2000 mPa·s. As used in filament winding process, the viscosity of resin should be in a certain range, 200–2000 mPa·s. If the viscosity of the resin system is overly high, it cannot completely penetrate into the fiber; if the viscosity value is too low, the resin will be easy to drop, and the fiber will contain little glue. Accordingly, it is considered that the resin system in 6 h displays good usability, which can satisfy the requirements of the wet filament winding process considering the viscosity characteristics of the resin system.

#### 3.1.4. The SEM Analysis of Nano-CSR(10 phr)/Nano-SiO_2_(2 phr)/Epoxy Resin System

**Figure 6:**
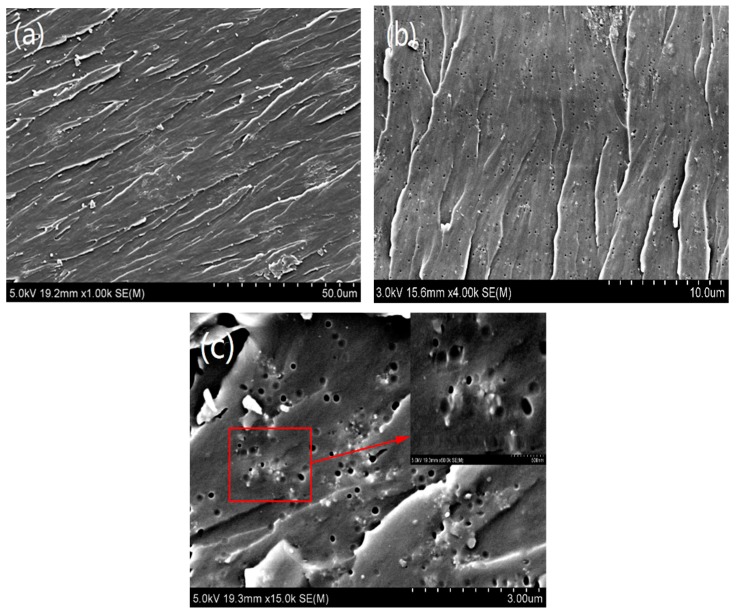
SEM analysis of a cross-section fracture of modified epoxy resin matrix: (**a**) ×1K; (**b**) ×4K; (**c**) ×15K.

The SEM analysis of cross section fracture of nanoparticles modified epoxy resin is shown in Figure 6, which reveal that the nanoparticles were uniformly distributed in the resin matrix, and the cross section displayed a fish-scale ductile fracture structure and rough surface. The roughness of the section suggested that the interaction between the nanoparticles and the resin matrix would generate more microcracks close to the particles and vary the direction, thereby forming a corrugated fish-scale structure and achieving toughening effect [16]. As shown in Figure 6, on the fracture surface of the resin, some CSR particles were separated from the cross-section, leaving a lot of holes, while most SiO_2_ particles remained embedded, which further indicated that the two kinds of particles exhibited different toughening mechanisms. The toughening mechanism of CSR particles was primarily cavitation theory. When the sample was subjected to external forces, due to the stress concentration of CSR particles, holes were generated between the interface with the matrix and itself. Therefore, when the resin was broken, CSR particles were separated from the resin matrix. While that of SiO_2_ particles was primarily riveted crack mechanism, the particles were tightly bound with the matrix and did not easily fall off.

### 3.2. Properties of NOL Ring Composite

#### 3.2.1. Mechanical Properties of NOL Rings

The mechanical properties of NOL ring samples are listed in Table 5. Tensile and shear strength of NOL ring samples are important parameters to characterize the properties of winding products. Table 4 suggests that the mechanical properties of NOL ring samples prepared by modified winding resin were enhanced compared with those before modification (CSR and SiO_2_ particles were not added). The tensile strength, Young’s modulus and interlaminar shear strength were enhanced by 14.0%, 4.56% and 18.9%, respectively. The results reveal that the modified resin can improve the properties of carbon fiber wound composites, and it helps enhance the overall mechanical properties of the product.

#### 3.2.2. SEM Analysis of NOL Ring Composite with Modified Epoxy Resin

**Figure 7:**
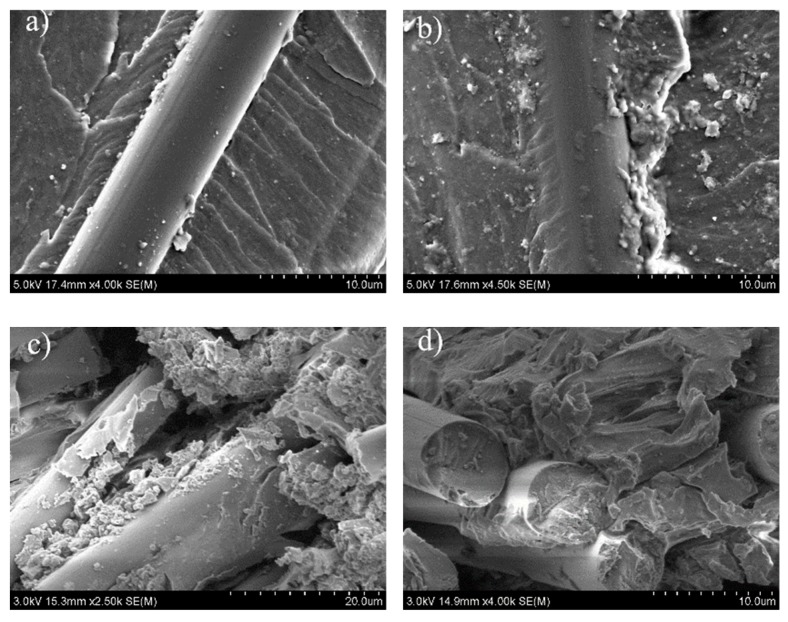
SEM analysis of NOL composite (10phr CSR/2phr SiO_2_): (**a**,**b**) SEM of interlaminar shear failure surface; (**c**,**d**) SEM of tensile fracture surface.

According to the results of scanning electron micrography of interlaminar shear failure surface (Figure 7a,b), the surface of the fiber was tightly coated by the matrix resin, the exposed fiber was reduced, and the fracture surface of the matrix displayed a fish-scale striation, which suggested that the modified resin matrix exhibited a good interface with the fiber.

Since the filament wound products are actually a laminated structure, there are no carbon fibers between the layers, when a composite undergoes an interlaminar shear loading, the interlaminar shear strength will depend on the strength of the resin matrix itself and the strength of the matrix–fiber interface [24,25]. According to the SEM of NOL rings failure surface, the interface was finely bonded. It is beneficial to improve the mechanical properties of composites. At the same time, the strength and toughness of modified resin were obviously improved, so the shear property of carbon fiber composites was also improved.

Likewise, the tensile fracture of NOL ring (Figure 7c,d) reveals that while considerable resins still adhere to the fiber surface, the gaps between carbon fibers also cover resins, the fracture of carbon fiber is relatively neat, and there is no fiber pulling out, which suggests that the fiber and resin exhibited good interface bonding properties. Thus, the modified resin can effectively transfer load and fully exploit the strength of carbon fiber. Above all, the addition of nanoparticles improved the toughness of the resin, which can inhibit the occurrence and expansion of the damage in the composite material to some extent, so as to improve the tensile strength of the composite material.

## 4. Conclusions

By adding CSR nanoparticles and nano-SiO_2_ particles, the epoxy resin was modified. It was found that when the additional amount was confined to a certain range, the CSR nanoparticles significantly improved the toughness and elongation of the resin, while the addition of nano-SiO_2_ particles could promote the enhancement of the strength and modulus of the resin. After the formula was optimized, 10 phr CSR nanoparticles and 2 phr nano-SiO_2_ particles were added, exerting a good synergetic modification effect. The modified resin tensile strength and modulus were 89 MPa and 3.5 GPa, while flexural strength and modulus reached 128 MPa and 3.2 GPa, respectively. The impact strength was 26.6 KJ·m^−2^, and the glass transition temperature reached 145.9 °C. The modified resin system also had good process performance with low viscosity and long service life. The viscosity was nearly 1000 mPa·s at 25 °C and service life was over 6 h. Modified epoxy resin enhanced the mechanical properties of carbon fiber reinforced wound composite. The tensile strength, Young’s modulus and interlaminar shear strength were enhanced by 14.0%, 4.56% and 18.9%, respectively, compared with the composite based on unmodified resin. In the meantime, the raw material cost of the resin system is low, the preparation process is simple, and so it is of practical implication to market application.

## Figures and Tables

**Table 1:** The major parameters of CSR particles.

Item	Parameter Indexes
Nuclear component	Butadiene rubber (BR) Polymethyl
Shell composition	methacrylate(PMMA)
Type of epoxy	Bisphenol A epoxy resin
Epoxy equivalent	301 (g/eq)
Core-shell rubber size	100 nm
Viscosity (50 °C)	25000 mPa·s
CSR active ingredient content	40 wt %

**Table 2:** The major parameters of nano-SiO_2_ particles.

Item	Parameter Indexes
Specific surface area (BET)	110 ± 20 m/g
Average particle size of primary particles	16 nm
carbon content	0.6–1.2 wt %
bulk density	50 g/L
SiO_2_ content (after burning)	99.8 wt %

**Table 3:** Test results of CSR nanoparticles modified epoxy resin.

Performance	CSR Particles, Addition				
	0	5 phr	10 phr	15 phr	20 phr
Tensile strength/MPa	65 ± 3	76 ± 3	82 ± 3	81 ± 4	78 ± 5
Young’s modulus/GPa	3.2 ± 0.1	3.1 ± 0.2	3.1 ± 0.1	2.9 ± 0.3	2.7 ± 0.3
Flexural strength/MPa	113 ± 3	115 ± 4	116 ± 3	118 ± 5	115 ± 4
Flexural modulus/GPa	3.0 ± 0.1	2.9 ± 0.2	2.9 ± 0.2	2.9 ± 0.2	2.8 ± 0.3
Impact strength/KJ·m^−2^	16.5 ± 3.5	20.5 ± 4.8	25.2 ± 5.1	28.5 ± 6.2	30.3 ± 6.3
Elongation at break/%	4.4 ± 0.3	4.9 ± 0.3	5.5 ± 0.3	5.0 ± 0.4	4.6 ± 0.4
Heat deflection temperature/°C	112 ± 1	111 ± 1	110 ± 1	108 ± 1	106 ± 1

**Table 4:** Test results of nano-SiO_2_ particles modified epoxy resin (with 10 phr CSR).

Performance	Nano-SiO_2_ Particles, Addition				
	0 phr	1 phr	2 phr	3 phr	5 phr
Tensile strength/MPa	82 ± 3	87 ± 4	89 ± 3	80 ± 4	73 ± 4
Young’s modulus/GPa	3.1 ± 0.2	3.2 ± 0.2	3.5 ± 0.2	3.6 ± 0.2	3.7 ± 0.3
Flexural strength/MPa	116 ± 4	120 ± 5	128 ± 4	125 ± 6	115 ± 6
Flexural modulus/GPa	2.9 ± 0.1	3.1 ± 0.2	3.2 ± 0.2	3.3 ± 0.2	3.5 ± 0.3
Impact strength/KJ·m^−2^	25.2 ± 5.2	26.1 ± 5.4	26.6 ± 5.9	24.0 ± 6.2	16.3 ± 7.5
Elongation at break/%	5.5 ± 0.2	5.2 ± 0.3	4.9 ± 0.3	4.5 ± 0.3	3.9 ± 0.3
Heat deflection temperature/°C	110 ± 1	115 ± 1	117 ± 1	118 ± 1	120 ± 1

**Table 5:** Mechanical properties of NOL ring composite.

Test Project	Tensile Strength/MPa	Young’s Modulus /GPa	Interlaminar Shear Strength/MPa
NOL ring composite with unmodified epoxy	1987.8 ± 80.3	72.3 ± 6.5	49.5 ± 5.7
NOL ring composite with modified epoxy	2266.7 ± 73.5	75.6 ± 7.4	58.9 ± 4.5
